# Hybrid Adaptive Cubature Kalman Filter with Unknown Variance of Measurement Noise

**DOI:** 10.3390/s18124335

**Published:** 2018-12-07

**Authors:** Yuepeng Shi, Xianfeng Tang, Xiaoliang Feng, Dingjun Bian, Xizhao Zhou

**Affiliations:** 1 College of Intelligent Manufacturing and Automation, Henan University of Animal Husbandry and Economy, Zhengzhou 450011, China; syp@hnuahe.edu.cn; 2 Information Technology Center, Zhejiang University, Hangzhou 310027, China; 3 College of Electrical Engineering, Henan University of Technology, Zhengzhou 450001, China; 4 School of Information Engineering, Zhengzhou University, Zhengzhou 450000, China; bdjbray@163.com; 5 School of Business, University of Shanghai for Science and Technology, Shanghai 200093, China; xizhaozhou@163.com

**Keywords:** nonlinear system, hybrid adaptive filtering, weighted fusion estimation, square-root cubature Kalman filter, information filter

## Abstract

This paper is concerned with the filtering problem caused by the inaccuracy variance of measurement noise in real nonlinear systems. A novel weighted fusion estimation method of multiple different variance estimators is presented to estimate the variance of the measurement noise. On this basis, a hybrid adaptive cubature Kalman filtering structure is proposed. Furthermore, the information filter of the hybrid adaptive cubature Kalman filter is also studied, and the stability and filtering accuracy of the filter are theoretically discussed. The final simulation examples verify the validity and effectiveness of the hybrid adaptive cubature Kalman filtering methods proposed in this paper.

## 1. Introduction

In recent decades, nonlinear filtering has been widely used in military and civil fields such as target tracking, navigation, positioning, and intelligent manufacturing [1,2]. The theory and method of nonlinear filtering has became one of the most important research issues in the signal processing field, and has attracted increasing attention from researchers.

There are two main kinds of nonlinear filtering methods. The representative of the first kind of nonlinear filter is the extended Kalman filter (EKF), which linearizes the system model by Taylor expansion, holds the first order term, and ignores the second- and higher-order terms. The second kind of nonlinear filtering approximates the statistics of the system state, with examples being the unscented Kalman filter (UKF) and the cubature Kalman filter (CKF). Due to the model error of the linearization of the nonlinear system, the accuracy of the EKF is slightly lower, even leading to filtering divergence. Based on the unscented transformation to approximate the statistics of the system state, UKF was presented in [3]. Further, the cubature Kalman filter algorithm was proposed by Ienkaran in [4]. The CKF algorithm uses a third-degree spherical-radial cubature rule based on a Gaussian filtering framework. The algorithm has higher numerical stability and a smaller amount of calculation. Its excellent performance has made it widely used in various nonlinear system scenarios. Many advantages of CKF have attracted scholars to conduct in-depth research on it, considering that the traditional nonlinear filters often need to overcome the filtering divergence caused by high-dimensional operational errors. Drawing on the idea of square root filtering in the Kalman filter, Ienkaram and Haykin proposed the square-root cubature Kalman filter (SCKF) [5], which further improved the accuracy and stability of filtering. However, the traditional nonlinear filtering method requires knowledge of the mathematical model and the prior statistical information of noise when in practical application. Additionally, the statistical characteristics of noise in actual systems are usually indeterminate, which leads to a decline in the filtering accuracy.

For the problem of unknown statistical characteristics of measurement noise in real applications [6,7], Sage and Husa [8,9] proposed an excellent Sage–Husa suboptimal unbiased maximum a posteriori (MAP) estimator. Many scholars also adopted adaptive filtering techniques to improve the performance of the estimation algorithm, such as sliding window method [10], fading factor adjustment (FFA) [11,12,13], maximum a posteriori (MAP) estimator [14,15], and the variational Bayesian (VB) method [16,17,18,19], etc. Different methods for estimating the statistical characteristics of system noise are usually designed under different estimation criterions. How to use these methods to estimate the statistical characteristics of system noise is still an open issue.

For a class of nonlinear stochastic systems with inaccurate or unknown measurement noise variance (i.e., the priori measurement noise variance is not a precise value), an adaptive filtering algorithm based on SCKF is designed in this paper. Firstly, a novel fusion approach is proposed to estimate the measurement noise variance on the basis of the MAP and VB methods. Then, we use FFA to adjust the part of the variance matrix of the SCKF algorithm to obtain a hybrid adaptive SCKF algorithm (HASCKF) and the corresponding information filter (recorded as HASCIF). This is beneficial to reduce the effects on the adaptive filtering algorithm performance that may be caused by the estimation deviation of noise fusion. At the same time, the performance of the adaptive filtering algorithm is analyzed from two aspects based on the established HASCKF algorithm: the stability of the adaptive filtering algorithm and the filtering accuracy.

This paper is organized as follows: Section 2 formulates the nonlinear stochastic system and describes the problem of the inaccuracy of the the measurement noise variance. In Section 3, a novel noise variance fusion estimation algorithm HASCKF and the corresponding information filter are proposed based on the idea of weighted fusion. In Section 4, two simulation examples are utilized to display and verify the performance of the proposed algorithms. Section 5 provides the conclusions of this work.

## 2. Problem Description

Considering a class of discrete nonlinear stochastic systems, the state space model is described as follows [1]:

(1) x(k+1)=f(x(k))+w(k),

(2) z(k)=h(x(k))+v(k),

where $x\left(k\right)\in R^{n}$ is the state of the target, $z\left(k\right)\in R^{m}$ is the measurement, $f:R^{n}\to R^{n}$ is the evolution process of the nonlinear state, and $h:R^{n}\to R^{m}$ is the corresponding nonlinear measurement mapping.

The process noise $w(k)\in R^{n}$ is a Gaussian white noise with zero means and variance Q(k).

The measurement noise $v(k)\in R^{m}$ is a Gaussian white noise with zero means and variance R(k).

**Hypothesis** **1.** *The process noise w(k) and measurement noise v(k) in the model are mutually statistically independent.*

**Hypothesis** **2.** *The initial state of the system is x(0), with mean $x_{0}$ and variance $P_{0}$, and it is uncorrelated with v(k) and w(k).*

**Hypothesis** **3.** *The process noise variance Q(k) is known, but the measurement noise variance is only with an inaccuracy prior state $R_{0}$.*

For the nonlinear system described in (1) and (2), assuming that at the time *k*, we have the optimal estimation $\hat{x}\left(k-1|k-1\right)$ and the square root matrix of the error variance matrix S(k−1|k−1). Then, the state estimate $\hat{x}\left(k|k\right)$ and the square root of the variance matrix S(k|k) can be calculated according to the standard SCKF algorithm.

For nonlinear systems with determined noise variance, the SCKF algorithm has better estimation performance. However, when the priori value of the measurement noise variance is inaccurate, the final estimation $\hat{x}\left(k|k\right)$ and S(k|k) will have large errors.

## 3. Hybrid Adaptive SCKF Algorithm (HASCKF)

In order to improve the adaptive filtering accuracy for an inaccurate modeling system with unknown measurement noise variance, and to let the estimated noise variance be closer to the true noise variance, in this paper, a novel noise variance fusion estimation algorithm HASCKF is proposed based on the idea of weighted fusion.

### 3.1. Estimation Method of Measurement Noise

**Theorem** **1.** *Assume that the measurement noise variance estimated by MAP and VB methods at time k are denoted as ${\hat{R}}^{1}\left(k\right)$ and ${\hat{R}}^{2}\left(k\right)$, respectively. Then, the weighted fusion estimation of the measurement noise variance ${\hat{R}}^{g}\left(k\right)$ is*

(3) $$\begin{array}{c} {\hat{R}}^{g}\left(k\right)={\hat{R}}^{1}\left(k\right)+\left[T_{1}\left(k\right)-T_{12}\left(k\right)\right][T_{1}\left(k\right)+T_{2}\left(k\right)-2T_{12}{\left(k\right)]}^{-1}\left[{\hat{R}}^{2}\left(k\right)-{\hat{R}}^{1}\left(k\right)\right], \end{array}$$

*where*

(4) $$\begin{array}{c} \left\{\begin{array}{c} T_{1}\left(k\right)={∥{\hat{R}}^{g}\left(k-1\right)-{\hat{R}}^{1}\left(k\right)∥}_{F}^{2},\\ T_{12}\left(k\right)=tr\left[\left({\hat{R}}^{g}\left(k-1\right)-{\hat{R}}^{1}\left(k\right)\right)({\hat{R}}^{g}\left(k-1\right)-{\hat{R}}^{2}{\left(k\right))}^{T}\right],\\ T_{2}\left(k\right)={∥{\hat{R}}^{g}\left(k-1\right)-{\hat{R}}^{2}\left(k\right)∥}_{F}^{2}. \end{array}\right. \end{array}$$

*In the above equation, ${∥\;\cdot \;∥}_{F}^{}$ represents the Frobenius norm of the matrix, and the initial value of the fusion estimation ${\hat{R}}^{g}\left(0\right)=R_{0}$.*

**Proof.**  The weighted fusion estimation of the measurement noise variance ${\hat{R}}^{g}\left(k\right)$ can be expressed as the following linear combination:

(5) $${\hat{R}}^{g}\left(k\right)=a_{1}\left(k\right){\hat{R}}^{1}\left(k\right)+a_{2}\left(k\right){\hat{R}}^{2}\left(k\right).$$

Under the condition $a_{1}\left(k\right)+a_{2}\left(k\right)=1$, we minimize the performance criterion:

$$J\left(k\right)=tr\left\{\;\left[(R\left(k\right)-{\hat{R}}^{g}\left(k\right)\right]{\left[(R\left(k\right)-{\hat{R}}^{g}\left(k\right)\right]}^{T}\right\}$$

From $a_{1}\left(k\right)+a_{2}\left(k\right)=1$, we can get $a_{1}\left(k\right)=\left(1-a_{2}\left(k\right)\right)$, then substituting it into formula (5), we obtain

(6) $${\hat{R}}^{g}\left(k\right)={\hat{R}}^{1}\left(k\right)+a_{2}\left(k\right)\left[{\hat{R}}^{2}\left(k\right)-{\hat{R}}^{1}\left(k\right)\right].$$

Then, the overall estimation error is

$$\begin{array}{c} {\tilde{R}}^{g}\left(k\right)=R\left(k\right)-{\hat{R}}^{g}\left(k\right)\\ \;\;\;=a_{1}\left(k\right){\tilde{R}}^{1}\left(k\right)+a_{2}\left(k\right){\tilde{R}}^{2}\left(k\right)\\ \;\;\;=\left[1-a_{2}\left(k\right)\right]{\tilde{R}}^{1}\left(k\right)+a_{2}\left(k\right){\tilde{R}}^{2}\left(k\right). \end{array}$$

So, we can get

(7) $$\begin{array}{c} J\left(k\right)=tr[{\tilde{R}}^{g}\left(k\right){\left({\tilde{R}}^{g}\left(k\right)\right)}^{T}]\\ \;\;\;\;={\left[1-a_{2}\left(k\right)\right]}^{2}tr\left[{\tilde{R}}^{1}\left(k\right){\left({\tilde{R}}^{1}\left(k\right)\right)}^{T}\right]+\left[1-a_{2}\left(k\right)\right]tr\left[{\tilde{R}}^{1}\left(k\right){\left({\tilde{R}}^{2}\left(k\right)\right)}^{T}\right]a_{2}\left(k\right)\\ \;\;\;+a_{2}\left(k\right)tr\left[{\tilde{R}}^{2}\left(k\right){\left({\tilde{R}}^{1}\left(k\right)\right)}^{T}\right]\left[1-a_{2}\left(k\right)\right]+a_{2}^{2}\left(k\right)tr\left[{\tilde{R}}^{2}\left(k\right){\left({\tilde{R}}^{2}\left(k\right)\right)}^{T}\right]. \end{array}$$

Let $\frac{\partial J(k)}{\partial a_{2}\left(k\right)}=0$, and after simplification, we can get

(8) $$\begin{array}{c} a_{2}\left(k\right)=\left[T_{1}\left(k\right)-T_{12}\left(k\right)\right]{\left[T_{1}\left(k\right)+T_{2}\left(k\right)-T_{12}\left(k\right)-T_{21}\left(k\right)\right]}^{-1}, \end{array}$$

where

(9) $$\left\{\begin{array}{c} T_{1}\left(k\right)=tr\left[{\tilde{R}}^{1}\left(k\right){\left({\tilde{R}}^{1}\left(k\right)\right)}^{T}\right]\\ \;\;\;\;\;=tr[\left(R\left(k\right)-{\hat{R}}^{1}\left(k\right)\right){\left(R\left(k\right)-{\hat{R}}^{1}\left(k\right)\right)}^{T}]\\ \;\;\;\;\;={∥R\left(k\right)-{\hat{R}}^{1}\left(k\right)∥}_{F}^{2},\\ T_{12}\left(k\right)=tr\left[{\tilde{R}}^{1}\left(k\right){\left({\tilde{R}}^{2}\left(k\right)\right)}^{T}\right]\\ \;\;\;=tr[\left(R\left(k\right)-{\hat{R}}^{1}\left(k\right)\right){\left(R\left(k\right)-{\hat{R}}^{2}\left(k\right)\right)}^{T}],\\ T_{21}\left(k\right)=tr\left[{\tilde{R}}^{2}\left(k\right){\left({\tilde{R}}^{1}\left(k\right)\right)}^{T}\right]\\ \;\;\;=tr[\left(R\left(k\right)-{\hat{R}}^{2}\left(k\right)\right){\left(R\left(k\right)-{\hat{R}}^{1}\left(k\right)\right)}^{T}]\\ \;\;\;=T_{12}\left(k\right),\\ T_{2}\left(k\right)=tr\left[{\tilde{R}}^{2}\left(k\right){\left({\tilde{R}}^{2}\left(k\right)\right)}^{T}\right]\\ \;\;\;\;\;=tr[\left(R\left(k\right)-{\hat{R}}^{2}\left(k\right)\right){\left(R\left(k\right)-{\hat{R}}^{2}\left(k\right)\right)}^{T}]\;\\ \;\;\;\;\;={∥R\left(k\right)-{\hat{R}}^{2}\left(k\right)∥}_{F}^{2}. \end{array}\right.$$

Since the measurement noise variance R(k) is uncertain, Equation (9) cannot be directly calculated. For this reason, we replace R(k) with fusion estimated value ${\hat{R}}^{g}\left(k-1\right)$ of the measurement noise variance at time (k−1). Obviously, the initial value satisfies ${\hat{R}}^{g}\left(0\right)=R_{0}$, then substituting it to Equations (9), (8), and (6), respectively, we can get Equations (3) and (4). □

**Note 1** In the above theorem, since the measurement noise variance R(k) is uncertain, we replace R(k) with fusion estimate ${\hat{R}}^{g}\left(k-1\right)$ of the measurement noise variance. This approximate substitution has certain rationality, especially for the case of constant noise variance and slowly varying noise variance. From Theorem 1, the physical meanings of $T_{1}\left(k\right)$ and $T_{2}\left(k\right)$ are equivalent to the estimated error variance of the two noise variance estimation methods, and $T_{12}\left(k\right)$ and $T_{21}\left(k\right)$ are similar to their cross-variance.

**Inference** **1.** *If we do not consider the correlation between the estimation error of noise variance, that is $T_{12}\left(k\right)=T_{12}\left(k\right)=0$, then the weighted fusion estimation of measurement noise variance ${\hat{R}}^{g}\left(k\right)$ is*

(10) $$\left\{\begin{array}{c} {\hat{R}}^{g}\left(k\right)={\left[T_{1}^{-1}\left(k\right)+T_{2}^{-1}\left(k\right)\right]}^{-1}T_{1}^{-1}\left(k\right){\hat{R}}^{1}\left(k\right)\\ \;\;\;\;\;+{\left[T_{1}^{-1}\left(k\right)+T_{2}^{-1}\left(k\right)\right]}^{-1}T_{2}^{-1}\left(k\right){\hat{R}}^{2}\left(k\right),\\ T^{-1}\left(k\right)=T_{1}^{-1}\left(k\right)+T_{2}^{-1}\left(k\right). \end{array}\right.$$

*It can be directly derived according to the principle of simple convex combination fusion [1].*

**Note 2** Obviously, the result of inference 1 is easy to generalize to the case where the number of noise variance estimators $N_{e}\geq 3$. Assume that the error variance of the ith estimator $T_{i}\left(k\right)={∥{\hat{R}}^{g}\left(k-1\right)-{\hat{R}}^{i}\left(k\right)∥}_{F}^{2}$. Assuming that the estimation errors of various estimation methods are not related to each other, the fusion estimation of noise variance is

(11) $$\left\{\begin{array}{c} {\hat{R}}^{g}\left(k\right)={\left[\sum_{j}^{N_{e}}T_{j}^{-1}\left(k\right)\right]}^{-1}\sum_{j}^{N_{e}}T_{j}^{-1}\left(k\right){\hat{R}}^{j}\left(k\right),\\ T_{g}^{-1}\left(k\right)=\sum_{j}^{N_{e}}T_{j}^{-1}\left(k\right), \end{array}\right.$$

where $T_{g}^{}\left(k\right)$ can be regarded as the error variance of noise variance fusion estimation. Since $T_{i}\left(k\right)\geq 0$, $T_{g}^{}\left(k\right)\leq T_{i}^{}\left(k\right)$, which indicates that the estimated noise variance after fusion is superior to that of any single noise variance estimator.

### 3.2. HASCKF Algorithm

**Figure 1:**
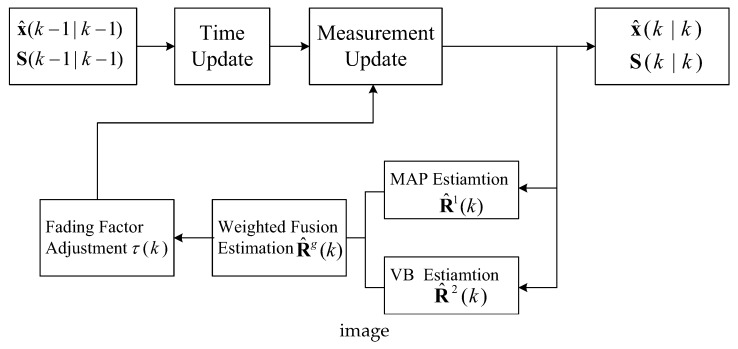
Principle block diagram of the HASCKF algorithm.

Based on Theorem 1, combined with the fading factor adjustment technique [11], we propose the hybrid adaptive SCKF algorithm (HASCKF). The principle block diagram is shown in Figure 1.

Firstly, the variance of measurement noise is estimated by the MAP estimator and VB method respectively. Then, the weighted fusion technique is introduced to fuse the two noise variance estimators. Finally, the variance matrix of the SCKF measurement update is adjusted by the fading factor adjustment technique to obtain the final state estimation and the root-mean square error (RMSE) variance matrix. The detailed implementation of HASCKF is described in the following Theorem 2.

**Theorem** **2.** *Consider a class of nonlinear system as described in (1) and (2). Under the condition of Hypotheses 1–3, if the optimal estimate $\hat{x}\left(k-1|k-1\right)$ and the square-root matrix of the estimation error variance S(k−1|k−1) have been obtained, the state estimate $\hat{x}\left(k|k\right)$ and the square-root matrix of the estimation error variance S(k|k) can be calculated according to the following steps:*

**Step 1: Time Update**

(12) $$x_{i}\left(k-1|k-1\right)=S\left(k-1|k-1\right)\xi_{i}+\hat{x}\left(k-1|k-1\right),$$

(13) $$x_{i}^{*}\left(k|k-1\right)=f\left(x_{i}\left(k-1|k-1\right)\right),$$

(14) $$\hat{x}\left(k|k-1\right)=\frac{1}{N_{x}}\sum_{i=1}^{N_{x}}x_{i}^{*}\left(k|k-1\right).$$

In (12)–(14), $i=1,2,\cdots ,N_{x}$, $N_{x}=2n$. The parameters $\xi_{i}$ are given below ($\varepsilon_{i}$ is an *n*-order unit vector):

ξi=NxNx22·εi,i=1,2,⋯,n,−NxNx22·εi−nx,i=n+1,n+2,⋯,Nx.

Calculate the square root of the variance matrix:

(15) $$S\left(k|k-1\right)=Tria(\left[X^{*}\left(k|k-1\right)\;\;\;\;\;\;\;S_{Q}\left(k\right)\right]),$$

where Tria(·) represents a triangular operation. $S_{Q}\left(k\right)$ represents the square-root of the new process noise variance Q(k), that is, $Q\left(k\right)=S_{Q}\left(k\right)S_{Q}^{T}\left(k\right)$, and

$$X^{*}\left(k|k-1\right)=\frac{1}{\sqrt{N_{x}}}\left[x_{1}^{*}\left(k|k-1\right)-\hat{x}\left(k|k-1\right)\;\right.,\;x_{2}^{*}\left(k|k-1\right)-\hat{x}\left(k|k-1\right),\left.\;\cdots \;,x_{N_{x}}^{*}\left(k|k-1\right)-\hat{x}\left(k|k-1\right)\right].$$

**Step 2: Measurement Update**

(**1**) According to Equations (16)–(18), we can calculate the predicted value $\hat{z}\left(k|k-1\right)$.

(16) $$x_{i}\left(k|k-1\right)=S\left(k|k-1\right)\xi_{i}+\hat{x}\left(k|k-1\right),$$

(17) $$z_{i}\left(k|k-1\right)=h\left(x_{i}\left(k|k-1\right)\right),$$

(18) $$\hat{z}\left(k|k-1\right)=\frac{1}{N_{x}}\sum_{i=1}^{N_{x}}z_{i}\left(k|k-1\right).$$

(**2**) The measurement noise variance ${\hat{R}}^{1}\left(k\right)$ of the MAP estimator is calculated by using Lemma 1 (19) or Lemma 2 (20).

**Lemma** **1** ([14])**.** *When the measurement noise variance is constant, the suboptimal MAP estimate $\hat{R}\left(k\right)$ of noise variance R(k) can be obtained by the recursive calculation:*

(19) $$\begin{array}{c} \hat{R}\left(k\right)=\frac{1}{k}[\left(k-1\right)\hat{R}\left(k-1\right)+\tilde{z}\left(k\right){\tilde{z}}^{T}\left(k\right)\;\\ -z\left(k|k-1\right)z^{T}\left(k|k-1\right)], \end{array}$$

*where $\tilde{z}\left(k\right)=z\left(k\right)-\hat{z}\left(k|k-1\right)$ is the residual vector of measurement. The initial value $\hat{R}\left(0\right)=R_{0}$.*

**Lemma** **2** ([15])**.** *When the measurement noise variance is time-varying, the suboptimal MAP estimate $\hat{R}\left(k\right)$ of noise variance R(k) can be obtained by the recursive calculation:*

(20) $$\begin{array}{c} \hat{R}\left(k\right)=\left[1-d\left(k-1\right)\right]\hat{R}\left(k-1\right)+d\left(k-1\right)[\tilde{z}\left(k\right){\tilde{z}}^{T}\left(k\right)\;\\ -z\left(k|k-1\right)z^{T}\left(k|k-1\right)], \end{array}$$

*where $d\left(k\right)=\left(1-b\right)/\left(1-b^{k+1}\right)$. b is the forgetting factor, and its value range is usually between 0.95 and 0.99.*

(**3**) Using the following Equation (21), combined with Equations (16)–(18), (22), (25), (26), and (23)–(32), we can iteratively compute the measurement noise variance estimation ${\hat{R}}^{2}\left(k\right)$ of the VB method.

Estimation of prediction parameters of measurement noise variance by VB method:

(21) $$\left\{\begin{array}{c} \zeta (k|k-1)=\rho \cdot \zeta (k-1),\\ \eta (k|k-1)=\rho \cdot \eta (k-1), \end{array}\right.$$

where “·” represents the point operation in Matlab. $\rho ={\left[\rho_{1},\cdots ,\rho_{m}\right]}^{T}$, $\zeta \left(k\right)={\left[\zeta_{1}\left(k\right),\cdots ,\zeta_{m}\left(k\right)\right]}^{T}$, $\eta \left(k\right)={\left[\eta_{1}\left(k\right),\cdots ,\eta_{m}\left(k\right)\right]}^{T}$. $\zeta_{i}\left(k\right)$ and $\eta_{i}\left(k\right)$ are two parameters of the inverse gamma distribution. $\rho_{i}\subset \left(0,1\right)\;$ is the predictive weighting factor. It reflects the degree of correlation between the noise at the last moment and the noise at the current moment. When the difference between the measurement noise variance at the last moment and the measurement noise variance at the current moment is small, a larger $\rho_{i}$ value should be used. Conversely, $\rho_{i}$ should take a smaller value.

The square-root of the variance matrix:

(22) $$S_{zz}\left(k|k-1\right)=Tria\left(\left[z\left(k|k-1\right)\;\;\;\;\;\;S_{R}\left(k\right)\right]\right),$$

where $S_{R}\left(k\right)$ represents the square root of measurement noise variance R(k) (namely, $R\left(k\right)=S_{R}\left(k\right)S_{R}^{T}\left(k\right)$) and

$$\begin{array}{c} z\left(k|k-1\right)\;\;=\frac{1}{\sqrt{N_{x}}}\left[z_{1}\left(k|k-1\right)\;\;-\hat{z}\left(k|k-1\right)\right.\\ \;\;\;\;\;\;\;z_{2}\left(k|k-1\right)\;\;-\hat{z}\left(k|k-1\right)\;\;\;\;\;\;\\ \;\;\;\;\;\;\;\left.\cdots \;\;\;\;\;\;\;z_{N_{x}}\left(k|k-1\right)\;\;-\hat{z}\left(k|k-1\right)\;\right], \end{array}$$

(23) $$P_{xz}\left(k|k-1\right)=x\left(k|k-1\right)z^{T}\left(k|k-1\right),$$

where

$$\begin{array}{c} x\left(k|k-1\right)=\frac{1}{\sqrt{N_{x}}}\left[x_{1}\left(k|k-1\right)-\hat{x}\left(k|k-1\right)\right.\\ \;\;\;\;\;\;\;x_{2}\left(k|k-1\right)-\hat{x}\left(k|k-1\right)\\ \;\;\;\;\;\left.\;\;\;\;\;\;\;\cdots \;\;\;\;\;\;\;x_{N_{x}}\left(k|k-1\right)-\hat{x}\left(k|k-1\right)\right]. \end{array}$$

Then, the VB method gets $\hat{R}\left(k\right)$ through *M* iterations:

Iterative initialization: let t=1, for a given number of iterations *M*, we have

(24) $$\left\{\begin{array}{c} \zeta \left(k\right)={\left[1/2,1/2,\cdots ,1/2\right]}^{T}+\zeta \left(k|k-1\right),\\ {\hat{x}}^{1}\left(k|k-1\right)=\hat{x}\left(k|k-1\right). \end{array}\right.$$

Calculating the estimate of the measurement noise variance:

(25) R^t(k)=diag(ηt(k)·ηt(k)·ζ(k)ζ(k)),

where diag(A) represents a diagonal matrix composed of matrix *A* diagonal elements.

Use Equations (16) and (32) to calculate the *t*-th iteration state estimate ${\hat{x}}^{t}\left(k|k\right)$ and the root of its mean square error matrix $S^{t}\left(k|k\right)$. If t<M, update the parameter $\eta^{t}\left(k\right)$.

(26) $$\begin{array}{c} \eta^{t}\left(k\right)=\eta \left(k|k-1\right)+{\left(z\left(k\right)-{\hat{z}}^{t}\left(k|k-1\right)\right)}^{\cdot 2}/2+diag\left\{P_{zz}^{t}\left(k|k\right)\right\}/2. \end{array}$$

Let t=t+1, ${\hat{x}}^{t}\left(k|k-1\right)={\hat{x}}^{t-1}\left(k|k\right)$, return to the beginning of the iterative.

When t=M, end the iteration, we can get

(27) $$\left\{\begin{array}{c} \eta \left(k\right)=\eta^{M}\left(k\right)\\ \hat{R}\left(k\right)={\hat{R}}^{M}\left(k\right) \end{array}\right.,\;\left\{\begin{array}{c} \hat{x}\left(k|k\right)={\hat{x}}^{M}\left(k|k\right)\\ S\left(k|k\right)=S^{M}\left(k|k\right) \end{array}.\right.$$

(**4**) Calculate the fusion estimation ${\hat{R}}^{g}\left(k\right)$ of the measurement noise variance according to Equation (3).

(**5**) According to Equations (28) and (29), $S_{zz}\left(k|k-1\right)$ and $P_{xz}\left(k|k-1\right)$ are adaptively adjusted using the FFA fading factor.

(28) Szz(k|k−1)=Tria[z(k|k−1)z(k|k−1)τ(k),τ(k)SR(k)]

(29) $$P_{xz}\left(k|k-1\right)=\frac{1}{\tau (k)}x\left(k|k-1\right)z^{T}\left(k|k-1\right),$$

where τ(k) is the adaptive factor, 0<τ(k)≤1, and it is calculated by the following Lemma 3.

**Lemma** **3** ([11])**.** *For nonlinear systems with unknown measurement noise variance, the adaptive fading factor is determined by the following equation:*

$$\tau \left(k\right)=\left\{\begin{array}{c} 1\;,\;\;\;\;\;\;\;\;\;\;\;\;tr[\tilde{z}\left(k|k-1\right){\tilde{z}}^{T}\left(k|k-1\right)]\leq tr\left[S_{zz}\left(k|k-1\right)S_{zz}^{T}\left(k|k-1\right)\right],\\ \frac{tr[S_{zz}\left(k|k-1\right)S^{T}\left(k|k-1\right)]}{tr[\tilde{z}\left(k|k-1\right){\tilde{z}}^{T}\left(k|k-1\right)]}\;,\;tr\left[\tilde{z}\left(k|k-1\right){\tilde{z}}^{T}\left(k|k-1\right)\right]>tr\left[S_{zz}\left(k|k-1\right)S_{zz}^{T}\left(k|k-1\right)\right]. \end{array}\right.$$

*In the above equation, tr denotes the trace of the matrix, $\tilde{z}\left(k|k-1\right)=z\left(k\right)-\hat{z}\left(k|k-1\right)$ is the measurement residual error vector.*

(**6**) The following Equations (30)–(32) are used to obtain the updated estimation $\hat{x}\left(k|k\right)$ and the root of its mean square error variance S(k|k).

(30) $$K\left(k\right)=\left(P_{xz}\left(k|k-1\right)/S_{zz}^{T}\left(k|k-1\right)\right)/S_{zz}\left(k|k-1\right),$$

where the symbol “/” indicates the matrix right divide operation (e.g., $A/B=AB^{-1}$)

(31) $$\hat{x}\left(k|k\right)=\hat{x}\left(k|k-1\right)+K\left(k\right)\left[z\left(k\right)-\hat{z}\left(k|k-1\right)\right],$$

(32) $$S\left(k|k\right)=Tria\left(\left[x(k|k-1)-K(k)z(k|k-1)\;\;K\left(k\right)S_{R}\left(k\right)\right]\right).$$

Proof: We can directly derive Theorem 1 and Lemma 3 by Lemma 1, omitted here.

**Note 3** Theorem 2 only shows the hybrid adaptive filtering algorithm when the number of noise variance estimators $N_{e}$ is 2. Obviously, when $N_{e}>2$, we first use various noise variance estimators to estimate ${\hat{R}}^{j}\left(k\right)$ and then calculate the fused estimate ${\hat{R}}^{g}\left(k\right)$ of the measurement noise variance according to Equation (11). In other words, only steps (2)–(4) in Theorem 2 need to be adjusted.

### 3.3. HASCIF Information Filter

Compared with traditional filtering, the information filter may not require prior information when it is initialized, and thus has better numerical performance. In addition, the use of an information filter to design a fusion algorithm is also simpler. In the information filter, the state estimate and its estimation error variance matrix are replaced by information vector and information matrix, respectively. Subsequently, we give its corresponding information filtering form (HASCIF) on the basis of HASCKF. According to the literature [20,21], we can obtain the implementation process of HASCIF as follows:

**Step 1:** Time update [20,21]

(33) $$\hat{y}\left(k|k-1\right)=Y\left(k|k-1\right)\hat{x}\left(k|k-1\right),$$

(34) $$Y_{}\left(k|k-1\right)=P_{}^{-1}\left(k|k-1\right)={\left[S\left(k|k-1\right)S^{T}\left(k|k-1\right)\right]}^{-1},$$

where $\hat{y}\left(k|k-1\right)$ and Y(k|k−1) are the predicted information vector and the predicted information matrix, respectively. $\hat{x}\left(k|k-1\right)$ and S(k|k−1) can be calculated according to Equations (14) and (15).

**Step 2:** Measurement update

(35) $$\begin{array}{c} \hat{y}\left(k|k\right)=Y\left(k|k\right)\hat{x}\left(k|k\right)\\ \;\;\;=\hat{y}\left(k|k-1\right)+Y\left(k|k-1\right)P_{xz}\left(k|k\right){\left[{\hat{R}}^{g}\left(k\right)\right]}^{-1}\left\{\tilde{z}\left(k|k-1\right)+P_{xz}^{T}\left(k|k\right)Y^{T}\left(k|k-1\right)\hat{x}\left(k|k-1\right)\right\}\\ \;\;\;\overset{\Delta }{=}\hat{y}\left(k|k-1\right)+\theta \left(k\right), \end{array}$$

(36) $$\begin{array}{c} \begin{array}{c} Y\left(k|k\right)=Y\left(k|k-1\right)+Y\left(k|k-1\right)P_{xz}\left(k|k\right){\left[{\hat{R}}^{g}\left(k\right)\right]}^{-1}\left(k\right)P_{xz}^{T}\left(k|k\right)Y^{T}\left(k|k-1\right)\\ \;\;\;\;\overset{\Delta }{=}Y\left(k|k-1\right)+\Theta \left(k\right). \end{array} \end{array}$$

In Equations (35) and (36), the information state vectors $\hat{y}\left(k|k\right)$ and Y(k|k) are the information vector and information matrix of the state estimate, respectively. θ(k) and Θ(k) are the information contribution vector and the information contribution matrix. ${\hat{R}}^{g}\left(k\right)$ is determined by formula (3), and the cross-variance matrix $P_{xz}\left(k|k\right)$ can be calculated by Formula (29).

### 3.4. Performance Analysis of HASCKF

References [22,23,24] proposed the bounded convergence theorem of the UKF algorithm, and Reference [14] extended its theorem to the adaptive cubature Kalman filter (ACKF). In this section, the CKF bounded convergence theorem proposed in Reference [14] and the Cramer–Rao lower bound (CRLB) [25] are used to analyze the convergence of the HASCKF algorithm.

#### 3.4.1. Stability Analysis

Assuming that the noise variances Q(k) and R(k) are known accurately, and considering the nonlinear systems (1), (2), and the standard CKF algorithm [4], the corresponding state error variance matrix can be written as [14,22,24]:

(37) $$\begin{array}{c} P\left(k|k-1\right)=x\left(k|k-1\right)x^{T}\left(k|k-1\right)+Q\left(k-1\right)\\ \;\;\;\;\;\;=A\left(k\right)P\left(k-1|k-1\right)A^{T}\left(k\right)+\delta P\left(k|k-1\right)+Q\left(k-1\right)\\ \;\;\;\;\;\;=A\left(k\right)P\left(k-1|k-1\right)A^{T}\left(k\right)+\Xi \left(k\right), \end{array}$$

(38) $$\begin{array}{c} P_{zz}\left(k|k-1\right)=z\left(k|k-1\right)z^{T}\left(k|k-1\right)+R\left(k\right)\\ \;\;\;\;=G\left(k\right)P\left(k|k-1\right)G^{T}\left(k\right)+\delta P_{zz}\left(k|k-1\right)+R\left(k\right)\\ \;\;\;\;=G\left(k\right)P\left(k|k-1\right)G^{T}\left(k\right)+\Sigma \left(k\right), \end{array}$$

(39) $$\begin{array}{c} K\left(k\right)=P\left(k|k-1\right)G^{T}\left(k\right)[G\left(k\right)P\left(k|k-1\right)G^{T}\left(k\right){+\Sigma \left(k\right)]}^{-1}, \end{array}$$

where

(40) $$\begin{array}{c} \delta P\left(k|k-1\right)=x\left(k|k-1\right)x^{T}\left(k|k-1\right)-A\left(k\right)P\left(k-1|k-1\right)A^{T}\left(k\right), \end{array}$$

(41) $$\begin{array}{c} \delta P_{zz}\left(k|k-1\right)=z\left(k|k-1\right)z^{T}\left(k|k-1\right)-G\left(k\right)P\left(k|k-1\right)G^{T}\left(k\right). \end{array}$$

Each correlation matrix is defined as follows:

(42) $$\left\{\begin{array}{c} A(k)=\beta (k)F(k),\\ B(k)=\alpha (k)H(k),\\ C(k)=I-K(k)\alpha (k)H(k), \end{array}\right.$$

(43) $$G\left(k\right)=\left\{\begin{array}{c} \alpha \left(k\right)H\left(k\right)\gamma^{T}\left(k\right),\;n\geq m,\\ \gamma^{T}\left(k\right)\alpha \left(k\right)H\left(k\right),\;n<m, \end{array}\right.$$

(44) $$\left\{\begin{array}{c} \Xi (k)=\delta P(k|k-1)+Q(k-1),\\ \Sigma \left(k\right)=\delta P_{zz}\left(k|k-1\right)+R\left(k\right), \end{array}\right.$$

where

$$F\left(k\right)={\left.\frac{\partial f}{\partial x}\right|}_{x=\hat{x}\left(k-1|k-1\right)},\;H\left(k\right)={\left.\frac{\partial h}{\partial x}\right|}_{x=\hat{x}\left(k|k-1\right)}.$$

α(k), β(k), and γ(k) are auxiliary diagonal matrixes. Refer to Reference [22] for specific meanings.

**Lemma** **4** ([14])**.** *Consider the nonlinear systems (1), (2), and the standard CKF algorithm. If ∀k≥0, both satisfy the following two assumptions:*

*(1) There are non-zero real numbers $a_{\min}$, $a_{\max}$, $b_{\max}$, $c_{\max}$, $g_{\min}$, and $g_{\max}$ existing to let the following formulas be established:*

(45) $$\left\{\begin{array}{c} a_{\min}^{2}I\leq A\left(k\right)A^{T}\left(k\right)\leq a_{\max}^{2}I,\;B\left(k\right)B^{T}\left(k\right)\leq b_{\max}^{2}I,\\ g_{\min}^{2}I\leq G\left(k\right)G^{T}\left(k\right)\leq g_{\max}^{2}I,\;C\left(k\right)C^{T}\left(k\right)\leq c_{\max}^{2}I,\\ \left[G(k)-B(k)\right]{\left[G\left(k\right)-B\left(k\right)\right]}^{T}\leq {\left(g_{\max}^{}-b_{\max}^{}\right)}^{{}^{2}}I. \end{array}\right.$$

*(2) There are positive real numbers $p_{\min}$, $p_{\max}$, $q_{\max}$, $r_{\max}$, $\Xi_{\min}$, $\Xi_{\max}$, and $\Sigma_{\min}$ existing to let the following forms be established:*

(46) $$\left\{\begin{array}{c} p_{\min}^{}I\leq P\left(k|k\right)\leq p_{\max}^{}I,\;Q\left(k\right)\leq q_{\max}^{}I,\\ R\left(k\right)\leq r_{\max}^{}I,\;\Xi \left(k\right)\leq \Xi_{\max}^{}I,\\ \Xi \left(k\right)>\Xi_{\min}^{}I,\;\Sigma \left(k\right)>\Sigma_{\min}^{}I, \end{array}\right.$$

*where*

$$\left\{\begin{array}{c} \Sigma_{\min}^{}=\max\left(\Sigma_{1}^{},\Sigma_{2}^{}\right),\\ \Sigma_{1}^{}=a_{\max}^{2}{\left(g_{\max}^{}-b_{\max}^{}\right)}^{2}\left(p_{\max}^{}+p_{\max}^{2}a_{\max}^{2}\Xi_{\min}^{-1}\right),\\ \Sigma_{2}^{}=b_{\max}^{2}\left(a_{\max}^{2}p_{\max}^{}+\Xi_{\max}^{}\right)-g_{\max}^{2}\left(a_{\min}^{2}p_{\min}^{}\Xi_{\min}^{}\right). \end{array}\right.$$

*Then, the standard CKF state estimation error will be mean-square bounded, that is, the algorithm is stable and convergent.*

**Note 4** Lemma 4 shows that the statistical characteristics of noise are closely related to the stability of the CKF algorithm. In addition, if Lemma 4 is established, the SCKF with known noise statistics is also stable and convergent. This is because the theoretical framework of SCKF is consistent with that of CKF. The mean square root matrix S(k|k−1) and S(k|k) in CKF are only used when transferring the error variance. The root mean square matrix in the SCKF algorithm is obtained through triangulation of the matrix, thus avoiding the filter divergence caused by the non-positive definite variance matrix in the numerical calculation, so it has better stability while ensuring the accuracy of estimation with CKF.

**Theorem** **3.** *If the standard SCKF algorithm is stable and convergent when the statistical characteristics of noise are known accurately, the introduction of the noise variance fusion estimator and the adaptive fade factor can ensure the stable convergence of the HASCKF algorithm.*

**Proof.**  When the measured noise variance matrix R(k) is inaccurate, other sufficient conditions in the bounded convergence theorem can be satisfied, but the condition (46) will be affected.

(a) First only consider the influence of the weighted fusion noise estimator. Let $\Delta R\left(k\right)={\hat{R}}^{g}\left(k\right)-R\left(k\right)$, then formula (40) can be rewritten as follows:

(47) $$\begin{array}{c} \overset{⌣}{\;\Sigma }\left(k\right)=\delta P_{zz}\left(k|k-1\right)+{\hat{R}}^{g}\left(k\right)\\ \;\;\;=\delta P_{zz}\left(k|k-1\right)+R\left(k\right)+\Delta R\left(k\right). \end{array}$$

When ${\hat{R}}^{g}\left(k\right)\geq R\left(k\right)$, then ΔR(k)≥0, and $\overset{⌣}{\;\Sigma }\left(k\right)$ will become larger. Obviously, condition (46) still holds and the HASCKF algorithm still converges steadily.

When ${\hat{R}}^{g}\left(k\right)<R\left(k\right)$, then ΔR(k)<0, and $\overset{⌣}{\;\Sigma }\left(k\right)$ will become smaller. Now, condition (46) may not be satisfied. However, the noise variance weighted fusion estimator ${\hat{R}}^{g}\left(k\right)$ estimates and corrects the inaccurate noise variance matrix in real time so that it gradually tracks the real value R(k), thus making ΔR(k)→0, $\overset{⌣}{\;\Sigma }\left(k\right)\to \Sigma \left(k\right)$. In this way, ΔR(k)≥0 is gradually satisfied to Equation (42) to ensure stable convergence of the HASCKF algorithm.

(b) Further consider the effect of fading factors. According to Theorem 2, the variance matrix for measuring residuals is

(48) $$\begin{array}{c} {\overset{⌣}{\;P}}_{zz}\left(k|k-1\right)=S_{zz}\left(k|k-1\right)S_{zz}^{T}\left(k|k-1\right)\\ \;\;\;\;\;\;\;\;=\frac{1}{\tau (k)}z\left(k|k-1\right)z^{T}\left(k|k-1\right)+{\hat{R}}^{g}\left(k\right). \end{array}$$

Then, Equation (41) is rewritten as:

(49) $$\begin{array}{c} \delta {\overset{⌣}{\;P}}_{zz}\left(k|k-1\right)=\frac{1}{\tau (k)}z\left(k|k-1\right)z^{T}\left(k|k-1\right)-G\left(k\right)P\left(k|k-1\right)G^{T}\left(k\right)\\ \;\;\;\;\;\;\;=\left[\frac{1}{\tau (k)}-1\right]z\left(k|k-1\right)z^{T}\left(k|k-1\right)+z\left(k|k-1\right)z^{T}\left(k|k-1\right)-G\left(k\right)P\left(k|k-1\right)G^{T}\left(k\right)\\ \;\;\;\;\;\;\;=\left[\frac{1}{\tau (k)}-1\right]z\left(k|k-1\right)z^{T}\left(k|k-1\right)+\delta P_{zz}\left(k|k-1\right)\\ \;\;\;\;\;\;\;\overset{\Delta }{=}\Delta P_{zz}\left(k|k-1\right)+\delta P_{zz}\left(k|k-1\right). \end{array}$$

Therefore, Equation (44) can be written as

(50) $$\begin{array}{c} \overset{⌣}{\;\Sigma }\left(k\right)=\delta {\overset{⌣}{\;P}}_{zz}\left(k|k-1\right)+{\hat{R}}^{g}\left(k\right)\\ \;\;\;=\delta P_{zz}\left(k|k-1\right)+R\left(k\right)+\Delta P_{zz}\left(k|k-1\right)+\Delta R\left(k\right)\\ \;\;\;\overset{\Delta }{=}\Sigma \left(k\right)+\Delta \Sigma \left(k\right). \end{array}$$

It is known from the definition of fading factor 0<τ(k)≤1, so we can get

(51) ΔPzz(k|k−1)=[11τ(k)τ(k)−1]z(k|k−1)zT(k|k−1)≥0.

Similar to the analysis in (a), when ΔR(k)≥0 and ΔΣ(k)≥0, it is obvious that condition (46) holds and the stability of the HASCKF algorithm remains. When ΔR(k)<0, if $\Delta P_{zz}\left(k|k-1\right)$ is large enough, ΔΣ(k)≥0 can still be satisfied, and condition (46) still holds, so the HASCKF algorithm converges steadily. If $\Delta P_{zz}\left(k|k-1\right)$ is not enough to guarantee ΔΣ(k)≥0, the introduction of the weighted fusion noise variance estimator can also make ΔR(k)→0, so that ΔΣ(k)≥0 is established stepwise to ensure stable convergence of the HASCKF algorithm.

In summary, the introduction of the noise variance fusion estimator and adaptive fading factor in the HASCKF algorithm can ensure the stable convergence of the algorithm. □

**Note 5** This theorem combined with Note 2 shows that the noise variance estimation based on weighted fusion is superior to the estimate of any single noise variance estimator. Therefore, the hybrid adaptive HASCKF estimation algorithm has better stability than the adaptive SCKF using a single noise variance estimation algorithm.

#### 3.4.2. Filtering Accuracy Analysis

There is a lower bound on the minimum variance unbiased estimator of the state of the nonlinear filtering algorithm. It is widely used to evaluate the performance of nonlinear estimation. In practice, the lower limit of Cramer–Rao Lower Bound (CRLB) is commonly used. Denote $X_{0:k}=\left\{x\left(0\right),x\left(1\right),\cdots ,x\left(k\right)\right\}$, $Z_{0:k}=\left\{z\left(0\right),z\left(1\right),\cdots ,z\left(k\right)\right\}$, and $p(Z_{0:k},X_{0:k})$ is the joint probability density of $\left(Z_{0:k},X_{0:k}\right)$. $\hat{x}\left(k\right)$ is the unbiased estimation of x(k). Then, CRLB is defined as [25]:

(52) $$P\left(k|k\right)\overset{\Delta }{=}E\left\{\left[x\left(k\right)-\hat{x}\left(k\right)\right]{\left[x\left(k\right)-\hat{x}\left(k\right)\right]}^{T}\right\}\geq J^{-1}\left(k\right),$$

where J(k) is the Fisher information matrix:

(53) $$J\left(k\right)=E\left[-\frac{\partial^{2}\ln p\left(Z_{0:k},X_{0:k}\right)}{\partial^{2}x^{2}\left(k\right)}\right].$$

**Theorem 4** Assume that the nonlinear filter is applied to system (1), (2). Then

(54) $$P\left(k|k\right)\geq J^{-1}\left(k\right),$$

where

(55) $$\begin{array}{c} J\left(k\right)={\left[Q\left(k-1\right)+F\left(k\right)J^{-1}\left(k-1\right)F^{T}\left(k\right)\right]}^{-1}+H^{T}\left(k\right)R^{-1}\left(k\right)H\left(k\right). \end{array}$$

**Proof.**  We use J(k) to denote the information matrix of state x(k). Then, the information matrix J(k) can be recursively calculated according to the following formula [26]:

(56) $$J\left(k\right)=D_{22}\left(k\right)-D_{21}\left(k\right)\left[J\left(k-1\right)+D_{11}\left(k\right)\right]{}^{-1}D_{12}\left(k\right),$$

where

(57) $$\left\{\begin{array}{c} D_{11}\left(k\right)=E\left\{-\Delta_{x(k-1)}^{x(k-1)}\ln p\left[x\left(k\right)|x\left(k-1\right)\right]\right\},\\ D_{12}\left(k\right)=E\left\{-\Delta_{x(k-1)}^{x(k)}\ln p\left[x\left(k\right)|x\left(k-1\right)\right]\right\},\\ D_{21}\left(k\right)=E\left\{-\Delta_{x(k)}^{x(k-1)}\ln p\left[x\left(k\right)|x\left(k-1\right)\right]\right\}\\ \;\;\;\;=D_{12}^{T}\left(k\right),\\ D_{22}\left(k\right)=E\left\{-\Delta_{x(k)}^{x(k)}\ln p\left[x\left(k\right)|x\left(k-1\right)\right]\right\}\\ \;\;\;\;\;\;\;+E\{-\Delta_{x(k)}^{x(k)}\ln p\left[z\left(k\right)|x\left(k\right)\right]\}, \end{array}\right.$$

where $\Delta_{x}^{y}=\nabla_{x}\nabla_{y}^{T}$ means the second-order operator. $\nabla_{x}=\frac{\partial }{\partial x}$ is the first-order operator. Because process noise and measurement noise are Gaussian white noise, we have

(58) $$\begin{array}{c} -\ln p[x(k)|x(k-1)]\\ =-\ln\left\{\frac{e^{\left\{-\frac{1}{2}{\left[x(k)-f(x(k-1))\right]}^{T}Q^{-1}\left(k-1\right)\left[x\left(k\right)-f\left(x\left(k-1\right)\right)\right]\right\}}}{\sqrt{2\pi }\left|Q(k-1)\right|}\right\}\\ =c_{1}+\frac{1}{2}{\left[x\left(k\right)-f\left(x\left(k-1\right)\right)\right]}^{T}Q^{-1}\left(k-1\right)\left[x\left(k\right)-f\left(x\left(k-1\right)\right)\right] \end{array}$$

(59) $$\begin{array}{c} -\ln p[z(k)|x(k)]\\ =-\ln\left\{\frac{e^{\left\{-\frac{1}{2}{\left[z(k)-h(x(k))\right]}^{T}R^{-1}\left(k\right)\left[z\left(k\right)-h\left(x\left(k\right)\right)\right]\right\}}}{\sqrt{2\pi }\left|R(k)\right|}\right\}\\ =c_{2}+\frac{1}{2}{\left[z\left(k\right)-h\left(x\left(k\right)\right)\right]}^{T}R^{-1}\left(k\right)\left[z\left(k\right)-h\left(x\left(k\right)\right)\right], \end{array}$$

where constant $c_{1}=\ln\left[\sqrt{2\pi }\left|Q(k-1)\right|\right]$, $c_{2}=\ln\left[\sqrt{2\pi }\left|R(k)\right|\right]$.

Substituting Equations (58) and (59) into Equation (57), then we can get

(60) $$\left\{\begin{array}{c} D_{11}\left(k\right)=E\left\{\left[\nabla_{x(k-1)}^{}f^{T}\left(x\left(k-1\right)\right)\right]Q^{-1}\left(k-1\right){\left[\nabla_{x(k-1)}^{}f^{T}\left(x\left(k-1\right)\right)\right]}^{T}\right\}\\ \;\;\;\;=F^{T}\left(k\right)Q^{-1}\left(k-1\right)F\left(k\right),\\ D_{12}\left(k\right)=E\{\left[\nabla_{x(k-1)}^{}f^{T}\left(x\left(k-1\right)\right)\right]Q^{-1}\left(k-1\right)\\ \;\;\;\;=F^{T}\left(k\right)Q^{-1}\left(k-1\right)\;=D_{21}^{T}\left(k\right),\\ D_{22}\left(k\right)=Q^{-1}\left(k-1\right)+E\left\{\left[\nabla_{x(k)}^{}h^{T}\left(x\left(k\right)\right)\right]R^{-1}\left(k\right){\left[\nabla_{x(k)}^{}h^{T}\left(x\left(k\right)\right)\right]}^{T}\right\}\\ \;\;\;\;=Q^{-1}\left(k-1\right)+H^{T}\left(k\right)R^{-1}\left(k\right)H\left(k\right). \end{array}\right.$$

(61) $$\begin{array}{c} J\left(k\right)=Q^{-1}\left(k-1\right)+H^{T}\left(k\right)R^{-1}\left(k\right)H\left(k\right)\\ \;\;\;\;-Q^{-1}\left(k-1\right)F\left(k\right)\times \left[J(k-1\right)+F^{T}\left(k\right)Q^{-1}{\left(k-1\right)F\left(k\right)]}^{-1}F^{T}\left(k\right)Q^{-1}\left(k-1\right). \end{array}$$

Equation (56) can be obtained by applying a matrix inversion lemma to Equation (61). □

## 4. Simulation

In this section, two numerical simulation examples are provided to display and verify the performance of the SACKF algorithm proposed in this paper, mainly including the following contents:

(1) For a class of inaccurate modeling with unknown measurement noise variance, we compare the performance difference between the weighted fusion estimator (referred to as WF-NE) and the single noise estimator (e.g., MAP estimation or VB estimation, respectively denoted as MAP-NE and VB-NE).

(2) For a class of inaccurate modeling with unknown measurement noise variance, we study the advantages and disadvantages of the HASCKF algorithm and the standard SCKF algorithm, and the equivalence between the HASCKF and HASCIF algorithms.

**Example** **1.** *This example is used to evaluate the performance of three kinds of noise estimators, MAP-NE, VB-NE, and WF-NE. Considering the following first-order nonlinear discrete dynamic system:*

(62) $$x\left(k\right)=0.5x\left(k-1\right)+\frac{0.2x(k-1)}{1+x^{2}\left(k-1\right)}+w\left(k\right),$$

(63) $$z\left(k\right)=\frac{x^{2}\left(k\right)}{20}+v\left(k\right),$$

*where w(k) and v(k) are mutually independent Gaussian white noise sequences. Assume that the initial value of the system state and the error variance matrix are*

(64) $$x_{0}=2\;\;,\;\;P_{0}=0.01,$$

*and the system state initial value $x_{0}$ is independent of the two noises. The process noise statistic Q(k)=0.001. In the following, simulation experiments are performed for two cases where the measurement noise variance R(k) is a constant and piecewise continuous function.*

(1) Situation 1: If the measurement noise variance is constant and R(k)=0.012. Assume that the imprecise measurement noise variance of the initial value ${\hat{R}}_{0}=0.04$. In the simulation, MAP-NE adopted the estimator described in Equation (19). The parameters of VB-NE were selected as follows: $\rho =1-e^{-5}$, ζ(0)=1, η(0)=0.04, M=1.

In order to compare the performance of various algorithms, we adopted the absolute error (AE) and the mean absolute error (MAE), which are calculated as follows:

(65) $$AE\left(k\right)=\left|y\left(k\right)-\hat{y}\left(k\right)\right|\;\;,\;\;MAE\left(k\right)=\frac{1}{N_{s}}\sum_{k=1}^{N_{s}}AE(k),$$

where y(k) and $\hat{y}\left(k\right)$ are the value to be estimated and the estimated value respectively. $N_{s}$ is the number of simulation steps. In this case, $N_{s}=1000$.

**Figure 2:**
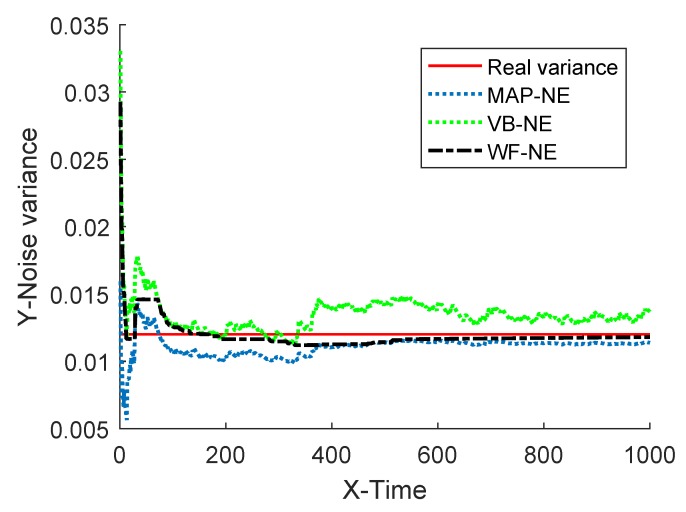
Estimation of measurement noise variance.

**Figure 3:**
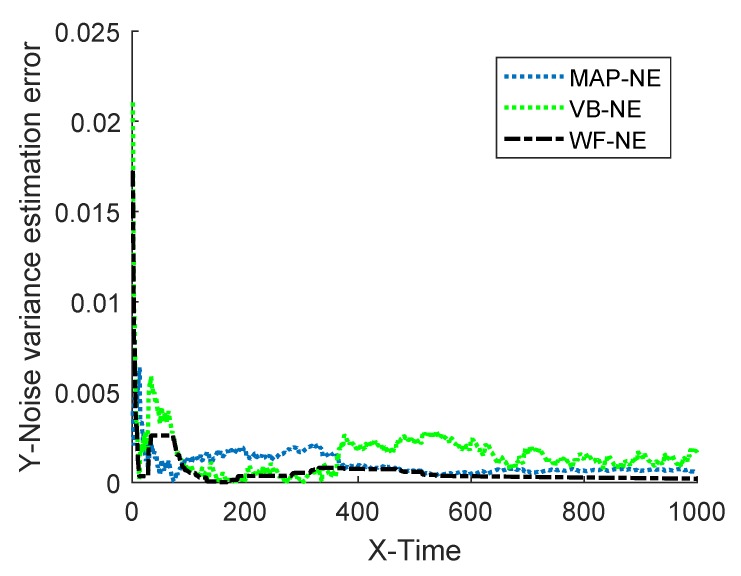
Absolute estimation error of measurement noise variance.

The estimated noise variance of the three kinds of noise estimators are shown in Figure 2 and Figure 3, and the estimation error is given in Table 1.

(2) Situation 2: If the measurement noise value is time varying, the true statistical characteristics meet the following formula:

(66) $$R\left(k\right)=\left\{\begin{array}{c} R\;\;0\leq k\leq 500\\ 2R\;\;\;501\leq k\leq 1000 \end{array}\right.,\;and\;R=0.012.$$

**Figure 4:**
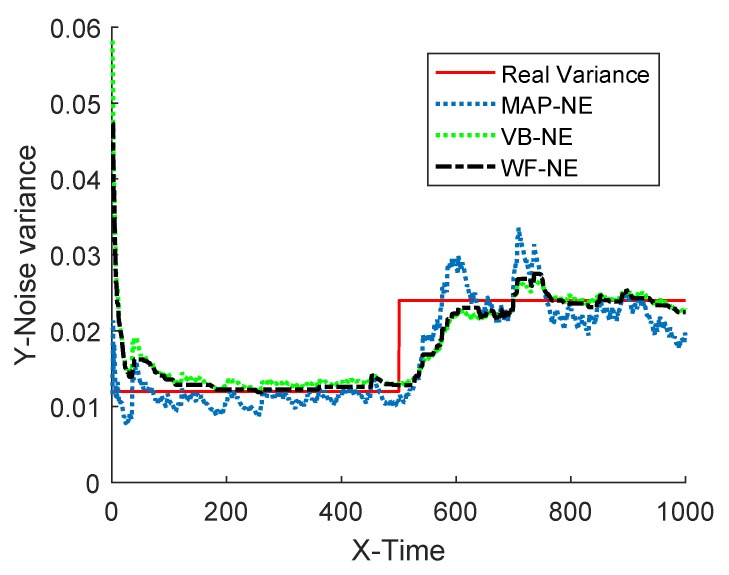
Estimation of measurement noise variance.

**Figure 5:**
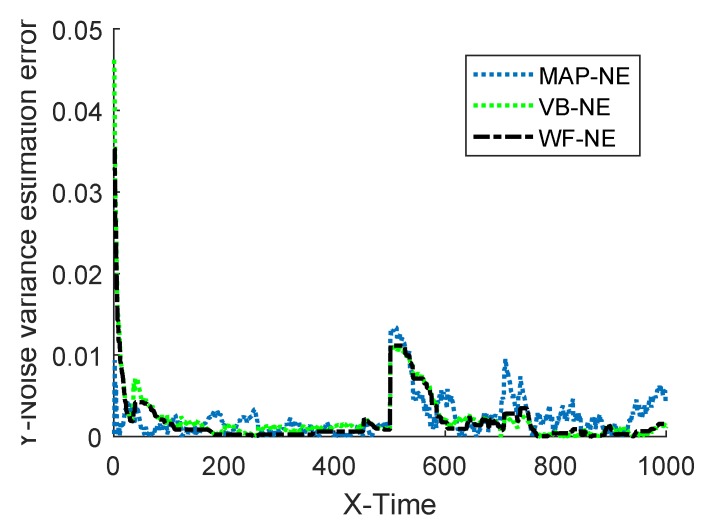
Absolute estimation error of measurement noise variance.

Assume that the known inaccurate initial value of the measurement noise variance is ${\hat{R}}_{0}=0.08$. In the simulation, MAP-NE adopted the estimator described in Equation (20), and the forgetting factor *b* was 0.98. The parameters of VB-NE were selected as $\rho =1-e^{-5}$, ζ(0)=1, η(0)=0.08, M=1. The estimation results of the three kinds of noise estimators on the measured noise variance are shown in Figure 4 and Figure 5, and the mean absolute errors of several algorithms are given in Table 2.

The simulation results of Example 1 show that the fusion estimator WF-NE proposed in Theorem 1 could obtain the best estimated result for either the constant unknown measurement noise variance or for the time-varying one, compared with the MAP-NE and VB-NE. It had the same result with the analysis conclusion in Note 2. In Situation 1, as shown in Figure 2 and Figure 3, compared with the fusion results of MAP-NE and VB-NE, the WF-NE had better estimates in most simulation steps. Due to the randomness of noise, not all the noise variance estimates obtained by WF-NE were better than the ones solved by other methods, especially for the estimation of the time-varying variance in Situation 2. As shown in Figure 3, the MAP-NE method had relatively large estimation errors in the time interval [160–360], and the VB-NE method had relatively large estimation errors in the time interval [390–800]. Nethertheless, the WF-NE could avoid large variation of the noise variance estimation error. Similarly, the general trend of the WF-NE method was best in Figure 4, although the MAP-NE method or the VB-NE method were best in some small time intervals. Although the CPU time cost of WF-NE (0.3725 for 1000 simulation steps) was larger than the other two methods (0.2188 for 1000 simulation steps), it was still acceptable. Meanwhile, the mean absolute errors of WF-NE were smaller than the other two methods, for boththe constant noise variance (in Situation 1) and for the time-varying variance (in Situation 2).

**Example** **2.** *This example is used to verify the pros and cons of the hybrid adaptive SCKF estimation algorithm HASCKF proposed by Theorem 2 and the standard SCKF algorithm described in Reference [4]. Assume that the target is moving in a uniform linear motion on a two-dimensional plane. The system state $x\left(k\right)={\left[x\left(k\right),\dot{x}\left(k\right),y\left(k\right),\dot{y}\left(k\right)\right]}^{T}$, where x(k) and y(k) are the position components in the east–north coordinate system. $\dot{x}\left(k\right)$ and $\dot{y}\left(k\right)$ are the corresponding velocity components, respectively. Then, the state equation can be described as:*

(67) $$x\left(k\right)=\left[\begin{array}{c} 1\;T\;0\;0\\ 0\;1\;0\;0\\ 0\;0\;1\;T\\ 0\;0\;0\;1 \end{array}\right]\cdot x\left(k-1\right)+w\left(k\right),$$

*where the sampling period T=0.5 s, the process noise w(k) is zero mean Gaussian white noise. Its statistical characteristic is Q(k)=0.1×diag(Q1,Q2), and Q1=T3T333T2T222T2T222T.*

*Consider a radar to track the target. The nonlinear measurement equation can be expressed as*

(68) $$z\left(k\right)=\left[\begin{array}{c} \sqrt{x^{2}\left(k\right)+y^{2}\left(k\right)}\\ \arctan\left(\frac{y(k)}{x(k)}\right) \end{array}\right]+v\left(k\right).$$

*The real statistical characteristic of the measuring noise $R\left(k\right)=diag\left\{{\left(4m\right)}^{2},{\left(0.1^{\circ }\right)}^{2}\right\}$. During the simulation, the simulation time was set as $N_{s}=100\;s$. Suppose that the inaccuracy initial value of the measurement noise variance $\hat{R}\left(0\right)=diag\left\{{\left(81m\right)}^{2},{\left(0.3^{\circ }\right)}^{2}\right\}$. MAP-NE adopts the estimator described in Equation (19), and the parameters of VB-NE were selected as $\rho ={\left[1-e^{-5},1-e^{-5}\right]}^{T}$, $\zeta \left(0\right)={\left[1,1\right]}^{T}$, $\eta \left(0\right)={\left[100,0.02\right]}^{T}$, M=1. The initial state estimate and the estimation error variance matrix are*

$\begin{array}{l} x_{0}={\left[10,000\;m,150\;m/s,15,000\;m,200\;m/s\right]}^{T}\\ P_{0}=diag\left\{{\left(100\;m\right)}^{2},{\left(14\;m/s\right)}^{2},{\left(100\;m\right)}^{2},{\left(15\;m/s\right)}^{2}\right\}. \end{array}$

**Figure 6:**
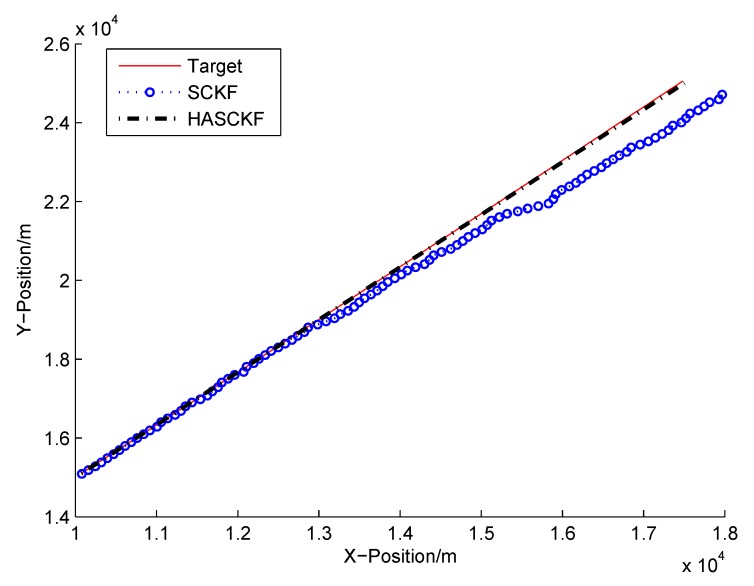
Target’s trajectory and tracking result of SCKF and HASCKF.

**Figure 7:**
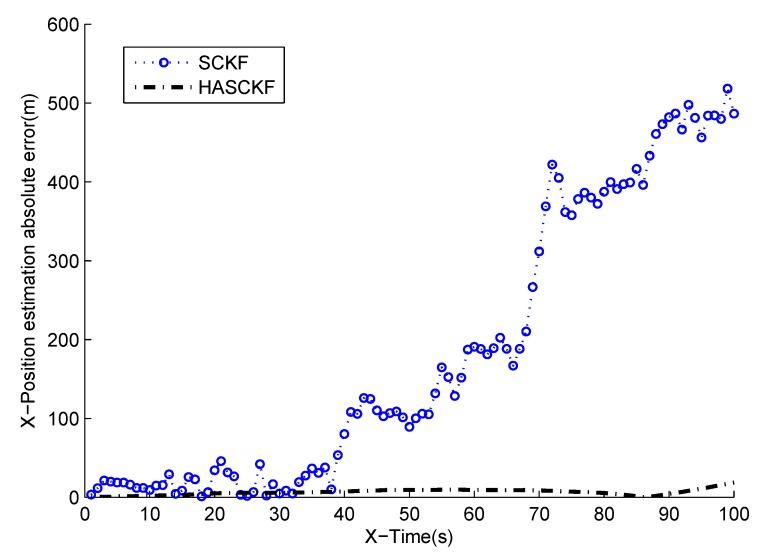
Absolute error curves of X-displacement.

**Figure 8:**
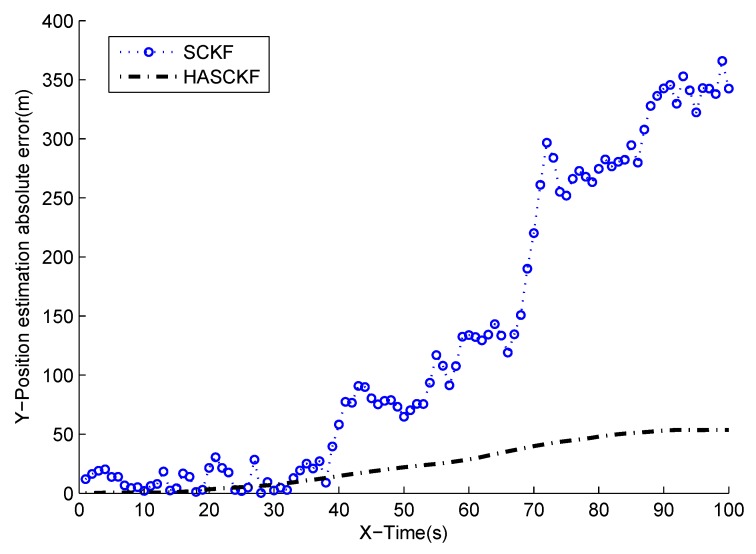
Absolute error curves of Y-displacement.

**Figure 9:**
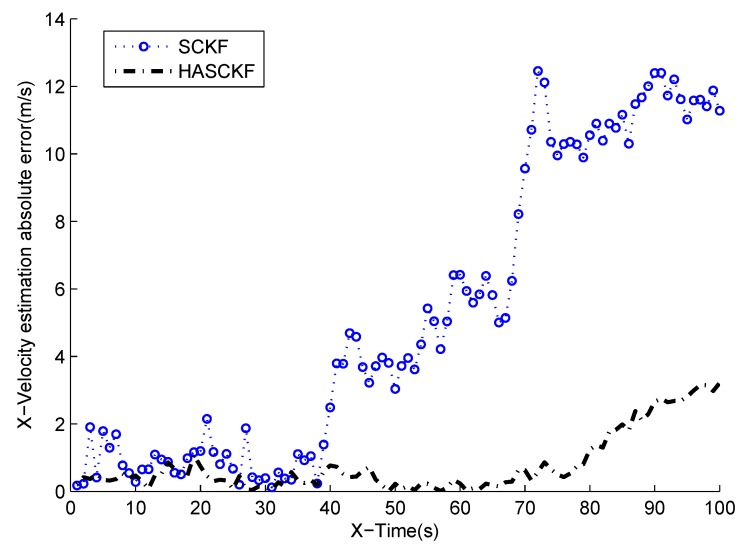
Absolute error curves of X-velocity.

**Figure 10:**
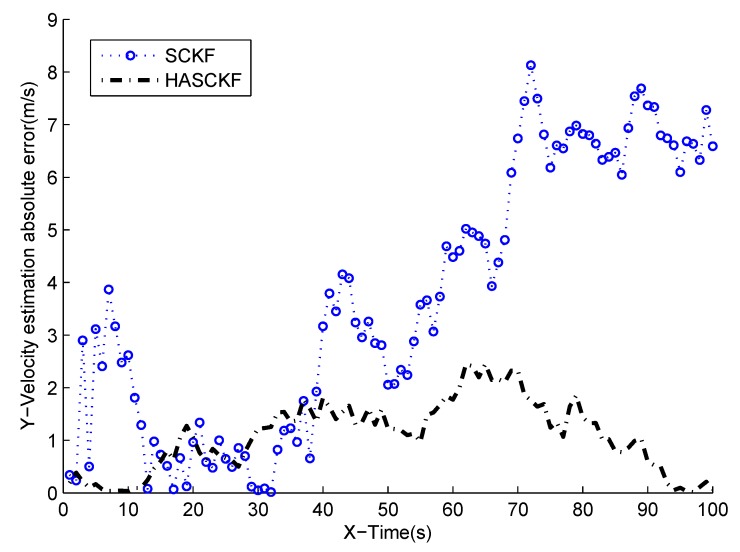
Absolute error curves of Y-velocity.

The estimated results of SCKF and HASCKF algorithms are shown in Figure 6, Figure 7, Figure 8, Figure 9 and Figure 10. The estimation error is given in Table 3.

From Figure 6 to Figure 10, it can be clearly seen that the standard SCKF estimation showed a large deviation after 40 s. However, HASCKF could still better estimate the state of the target. This is because the standard SCKF adopts an inaccurate prior noise variance $\hat{R}\left(0\right)$, while the HASCKF proposed in this paper estimates and corrects the inaccurate measurement noise variance, thus ensuring the accuracy and stability of the algorithm. As analyzed in Example 1, due to the randomness of noise, not all the noise variance estimates obtained by WF-NE were better. Therefore, the estimates of some components of the state obtained by SCKF were better than the one solved by HASCKF at some time in the interval [10 s, 40 s]. However, the general tend of the proposed HASCKF was better than SCKF, as shown in Figure 6, Figure 7, Figure 8, Figure 9 and Figure 10.

## 5. Conclusions

In this paper, aiming at the filtering problem caused by the inaccurate measurement noise variance in real systems, a weighted fusion estimation method is designed and a class of hybrid adaptive filtering structures is proposed, based on different measurement noise variance estimators. The stability and the filtering accuracy of the hybrid adaptive filter are analyzed and discussed theoretically. The simulation results showed that the algorithm had excellent accuracy and stability.

The work that can continue to be studied in the future includes further study on the research and design of fusion methods based on hybrid adaptive filtering in nonlinear centralized fusion framework and distributed fusion framework, research on functional equivalence among various fusion algorithms, and so on.

## Figures and Tables

**Table 1:** The mean absolute error of three noise estimation algorithms in Situation 1.

Algorithm	MAP-NE	VB-NE	WF-NE
Mean absolute error	0.0010	0.0015	0.0005
CPU time cost	0.2188	0.2188	0.3725

**Table 2:** The mean absolute error of three noise estimation algorithms in Situation 2.

Algorithm	MAP-NE	VB-NE	WF-NE
Mean absolute error	0.0024	0.0022	0.0019

**Table 3:** The mean absolute error of two algorithms.

Mean Absolute Error	Algorithms	
	SCKF	HASCKF
X-Position (m)	182.9477	6.4784
X-Velocity (m/s)	5.2113	0.7898
Y-Position (m)	129.5228	24.6147
Y-Velocity (m/s)	3.6766	1.0834
